# Characterizing T Cells in SCID Patients Presenting with Reactive or Residual T Lymphocytes

**DOI:** 10.1155/2012/261470

**Published:** 2012-11-20

**Authors:** Atar Lev, Amos J. Simon, Luba Trakhtenbrot, Itamar Goldstein, Meital Nagar, Polina Stepensky, Gideon Rechavi, Ninette Amariglio, Raz Somech

**Affiliations:** ^1^Cancer Research Center, Chaim Sheba Medical Center, Sackler Faculty of Medicine, Tel Aviv University, 69978 Tel Aviv, Israel; ^2^Hematology Laboratory, Chaim Sheba Medical Center, Sackler Faculty of Medicine, Tel Aviv University, 69978 Tel Aviv, Israel; ^3^Pediatric Immunology Service of Edmond and Lily Safra Children's Hospital, Chaim Sheba Medical Center, Sackler Faculty of Medicine, Tel Aviv University, 69978 Tel Aviv, Israel; ^4^Department of Pediatric Hematology-Oncology, Hadassah Medical Center, Hadassah Hebrew University, 91120 Jerusalem, Israel

## Abstract

*Introduction*. Patients with severe combined immunodeficiency (SCID) may present with residual circulating T cells. While all cells are functionally deficient, resulting in high susceptibility to infections, only some of these cells are causing autoimmune symptoms. *Methods*. Here we compared T-cell functions including the number of circulating CD3^+^ T cells, *in vitro* responses to mitogens, T-cell receptor (TCR) repertoire, TCR excision circles (TREC) levels, and regulatory T cells (Tregs) enumeration in several immunodeficinecy subtypes, clinically presenting with nonreactive residual cells (MHC-II deficiency) or reactive cells. The latter includes patients with autoreactive clonal expanded T cell and patients with alloreactive transplacentally maternal T cells. *Results*. MHC-II deficient patients had slightly reduced T-cell function, normal TRECs, TCR repertoires, and normal Tregs enumeration. In contrast, patients with reactive T cells exhibited poor T-cell differentiation and activity. While the autoreactive cells displayed significantly reduced Tregs numbers, the alloreactive transplacentally acquired maternal lymphocytes had high functional Tregs. *Conclusion*. SCID patients presenting with circulating T cells show different patterns of T-cell activity and regulatory T cells enumeration that dictates the immunodeficient and autoimmune manifestations. We suggest that a high-tolerance capacity of the alloreactive transplacentally acquired maternal lymphocytes represents a toleration advantage, yet still associated with severe immunodeficiency.

## 1. Introduction

Severe combined immunodeficiency (SCID) is typically characterized by significantly low number and/or defective function of T and B cells. In some cases, T cells may present, as a result of residual autologous cells or transplacentally acquired maternal lymphocytes [1]. Residual autologous T cells are usually emerging from partial thymic maturation impairment such as in the case of Major histocompatibility complex class II (MHC-II) deficiency. MHC-II molecules drive the development, activation, and homeostasis of CD4^+^ T-helper cells. It is thus not surprising that the absence of MHC-II expression results in a severe primary immunodeficiency disease. Yet, the residual cells in MHC-II deficient patients are considered as nonreactive; therefore patients typically do not display significant autoimmune phenomena. Although immunity is extensively impaired in such cases, regulatory tolerance mechanisms are not known to be affected [2]. Moreover, while the mainstay of the diagnosis of MHC-II deficiency is the absence of constitutive and inducible expression of MHC-II molecules on all cell types, other tests for T-cell function are less informative in such patients. In contrast, patients with self-reactive cells have significant autoimmune features in addition to their clinical and molecular immunodeficient state. The origin of the reactive cells in such patients are either thymic release of T-cells that expand at the periphery or transplacentally transfers of maternal T lymphocytes. These cells expand in the periphery, causing tissue infiltration and damage due to breakdown of both central (e.g., autoimmune regulator, AIRE protein dysfunction) and peripheral (FOXP3^+^ deficiency) tolerance mechanisms [3]. For example, Omenn syndrome, a typical case of impaired T-cell differentiation with abnormal self-reactive cells, is invariably characterized by autoimmune features such as generalized scaly exudative erythroderma, enlarged lymphoid tissues, and peripheral expansion of oligoclonal T-cells, in addition to increased susceptibility for severe infections [3, 4]. The suggested mechanism for this phenomenon is the possible inability of the thymus to delete these abnormal clones due to compromise of both central and peripheral tolerance mechanisms [5]. A distinctive feature of SCID patients, which sometimes can clinically resemble Omenn, [6] is the presence of alloreactive cells originated from transplacentally maternal T lymphocytes. The maternal placenta, an incomplete bidirectional barrier, allows transfer of maternal cells to occur in healthy neonates. Immunocompetent newborns can rapidly reject the HLA-mismatched maternal cells by effective T-cell immunity. In contrast, SCID patients fail to eliminate these cells and T-cell engraftment was reported in as many as 40% of them [7]. Immunologic characterization of these cells and their advantage of passing the placenta and surviving, compared to other maternal T cells, have not been investigated in depth. In the minority of cases these cells were found to have a normal phenotype with some degree of *in vivo* activation, as shown by the expression of MHC class II molecules and/or the IL-2 receptor [8]. Moreover, maternal engraftment provided the required immune competence and resulted in prolonged survival in rare cases of SCID [9]. In most cases, however, maternal T cells have been described as clonal cells [10], suggestive of either transplacental passage of a very small selected number of T cells or secondary expansion of alloreactive clones in the host. Transplacentally acquired maternal T lymphocytes and the autoreactive cells seen in Omenn phenotype have many clinical and laboratory features in common, including atypical skin eruption, hepatosplenomegaly, eosinophilia, elevated IgE levels, pattern of TH2 cytokines, lack of T-cell activity, and a restricted repertoire of the T cell receptor [11]. These cells do not provide enough immunity and may clinically be symptomatic, attacking the patient's organs. However, in contrast to Omenn patients where symptoms are typically severe, clinical findings associated with the transplacentally acquired maternal T lymphocytes are usually mild, with up to 60% being asymptomatic or mild symptomatic graft-versus-host disease (GVHD) [12]. The reason for this discrepancy is not clearly understood. In the current study, we have analyzed T-cell function, thymic capacity, and regulatory T cells (Tregs) enumerations in various severe immunodeficiency patients presenting with different origins of their T cells, hypothesizing that different severe immunodeficiency subtypes have different characterization of these cells, in correlation with the clinical features in each distinct subtype.

## 2. Materials and Methods

### 2.1. Patients

Six patients with clinical phenotypes suggestive of severe immunodeficiency, with or without Omenn features, were studied. The Institutional Review Board (Sheba Medical Center, Tel Hashomer) approved this study and a written informed consent was obtained from all parents of study's participants.

### 2.2. Immune Work Up

Cells surface markers of peripheral blood mononuclear cells (PBMCs), lymphocyte proliferative response to mitogens, T-cell receptor variable *β* (TCR V*β*) expression and the amount of signal joint (sj) T-cell receptor excision circles (TRECs) were determined as previously described [13]. To estimate TREC copies, we compared the amplification Ct value in a given sample with a standard curve obtained from PCRs performed with 10-fold serial dilutions of an internal standard. In 40 healthy age-matched control samples where immunodeficiency was excluded, TREC copies were >400.

### 2.3. Cell Isolation and Analysis of Treg Cells

PBMCs were obtained by density gradient centrifugation on Histopaque 1077 (Sigma). The mouse mAbs against various human-cell surface markers used were as follows: CD3-FITC, CD4-FITC, CD4-PE, CD4-APC, CD25-APC (all obtained from BD Pharmingen), CD25-PE (Miltenyi Biotec), and the 236A/E7 mouse anti-hFOXP3-APC mAbs (eBioscience). The isotype-matched control mAbs were all purchased from BD Pharmingen. For detection of forkhead box P3 (FOXP3), the cells were fixed/permeabilized using the eBioscience FOXP3 staining buffer set, according to the manufacturer's protocol (eBioscience). Cell samples were analyzed on a FACSCalibur using the Cellquest software. CD4 or CD8 positive T cells were isolated from PBMCs by positive selection with CD4 or CD8 microbeads (Milteny Biotec). IFN*γ* and IL-2 cytokine detections were used to verify the presence of Tregs. Briefly, T cells were reactivated with 20 ng/mL PMA and 0.8 *μ*M ionomycin (Sigma) in the presence of monensin 2 *μ*g/mL for 5 h (GolgiStop from BD Biosciences). Thereafter, the cells were fixed, permeabilized and stained for FOXP3 (236A/E7-APC) with the eBioscience Kit. In addition, the cells were stained with CD4-FITC and for cytokines with anti-IFN*γ*-PE, IL-2-PE (from BD Biosciences).

### 2.4. Visualization of Engrafted Maternal T Cells

The patients' lymphocytes were visualized by a multiparametric cell-scanning system (Duet, BioView Ltd., Rehovot, Israel) for detecting the presence of transplacentally acquired maternal T lymphocytes as previously described [14]. The system combines morphological and fluorescence *in situ* hybridization (FISH) analyses of the same cell, thereby enhancing the specificity of pathological cell detection.

## 3. Results

### 3.1. Patients

Six patients, all presented during infancy, were included in this study. The clinical, immunologic, and molecular features of the patients are listed in Table 1 and were consisted with a phenotype of classical SCID with maternal-fetal transfusion (Pt1, Pt2), SCID-Omenn (Pt3, Pt4) or the combined immunodeficiency (CID) MHC II deficiency (Pt5, Pt6). Patients 1, 3, and 4 were found to have mutations in the RAG2 gene including G156V, G35V, and G95V+E480X protein substitutions, respectively. Patient 2 was found to have the common gamma chain (*γ*_c_) deficiency due to G68L mutation. In addition to the classical immunodeficiency clinical phenotypes (e.g., failure to thrive, recurrent infections) patients 3 and 4 had severe Omenn symptoms, including diffuse erythrodermia, alopecia, lymphadenopathy, and enlarged liver and spleen. In contrast, patients 1 and 2 had only mild diffuse skin eruption and initially were misdiagnosed as having mild Omenn phenotype. Patients 5 and 6 had no symptoms suggestive of Omenn and their cell HLA-DR expression was undetectable, suggestive of MHC-II deficiency.

### 3.2. Visualization of Maternal Engraftment

**Figure 1:**
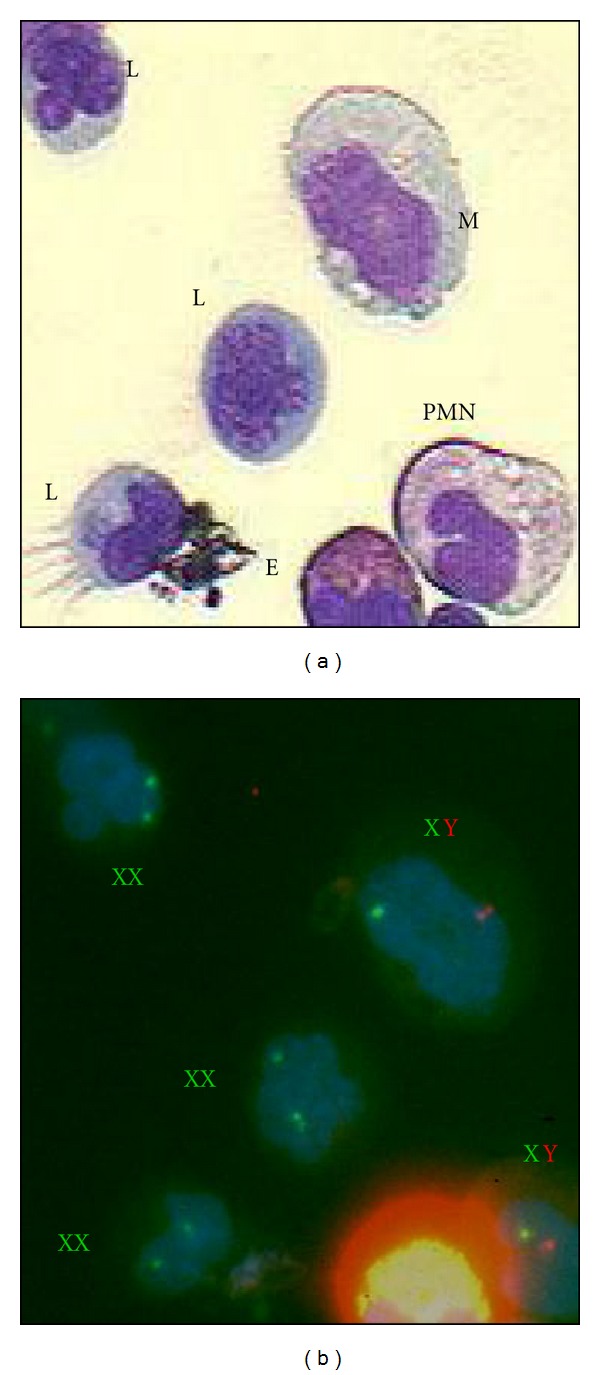
Visualization of the transplacentally acquired maternal cells. Combined morphological and FISH analysis confirmed the presence of transplacentally acquired maternal lymphocytes in patient 1 using the X and Y chromosomes probes. On the left (a), cells stained with Giemsa, and on the right (b) the same cells with FISH using dual-color XY DNA probe. XY genotype shows one green and one red FISH signals; XX genotype—two green signals. L = lymphocyte, YL = young lymphocyte, and PMN = polymorphonuclear cell.

Combined morphological and FISH studies were used in all patients to examine the presence of transplacentally acquired maternal lymphocytes (Table 1). In patients 1 and 2, all lymphocytes were of maternal origin while other hematopoietic cells were of the patient's origin (representation of patient 1 is given in Figure 1), thereby excluding the possibility of the presence of autoreactive or residual endogenous T cells. Based on this finding the patients' symptoms were suspected to be secondary to GVHD. In contrast, maternal engraftment was undetectable in patients 5 and 6, or negligible in patients 3 and 4, suggesting the presence of either residual cells (Pt5 and Pt6) or autoreactive cells (Pt3 and Pt4).

### 3.3. Immunologic Studies

**Figure 2:**
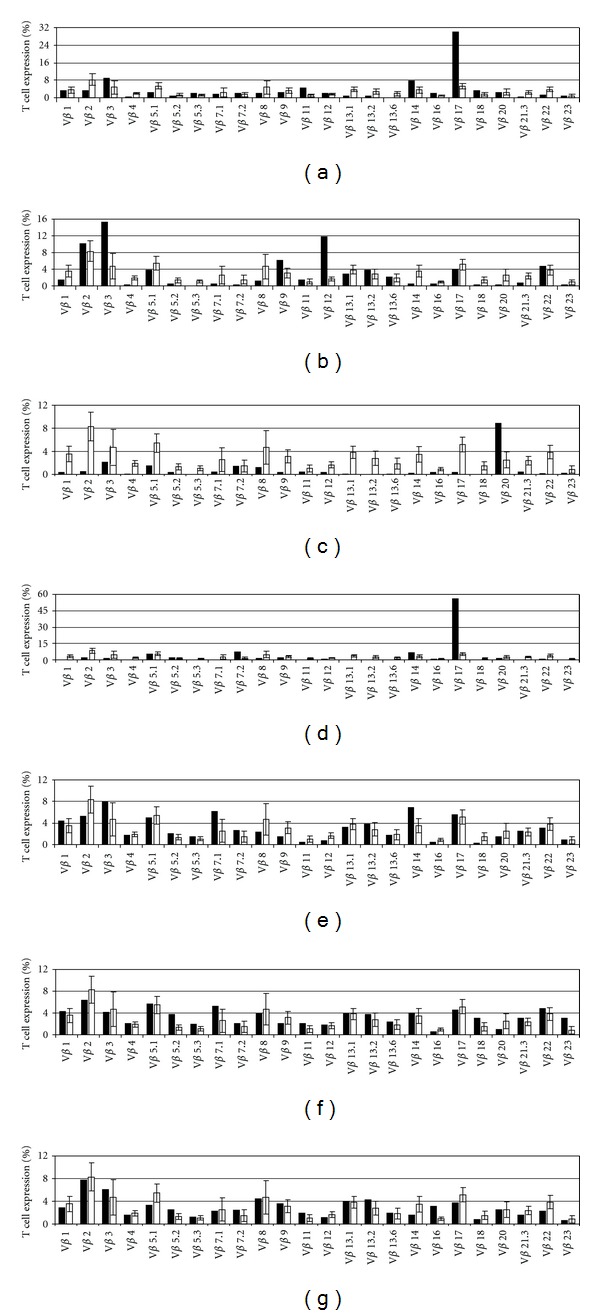
T-cell receptor (TCR) V*β* repertoire. Relative expression levels of 24 different TCR V*β* families in CD3^+^ cells (black bars) of pt1 (a), pt2 (b), pt3 (c), pt4 (d), pt5 (e), pt6 (f), and the mother of pt1 (g) compared with the relative expression of normal healthy controls (white bars) were obtained by FACS analyses. Normal control values were obtained using the IOTest Beta Mark TCR V*β* Repertoire Kit (Beckman Coulter).

All patients had peripheral CD3^+^ T lymphocytes. Four of them (Pt1, Pt2, Pt3, and Pt4) had skin erythrodermia and remarkable eosinophilia (Table 1). While 3 patients (Pt1, Pt3, and Pt4) were found to have no B lymphocytes, as could be expected in patients with the RAG2 deficiency, only patient 2 had no NK lymphocytes due to a genetic defect in the common *γ*_c_. Patients 5 and 6 had reduced CD4^+^ T lymphocytes with inverted CD4/CD8 ratio and subsequent measurement of HLA-DR revealed no expression at all. These findings were consistent with MHC-II deficiency. *In vitro* T-lymphocyte responses were significantly reduced in the patients with reactive T cells following phytohemagglutinin and anti-CD3 stimulations (3.8%–6.5% and 1.9%–31.5% of controls, resp.) and only slightly reduced in the MHC-II patients 5 and 6 (46.9%–94.8% and 32.8%–41.3% of controls, resp., Table 1). Similarly, the amount of recent thymic emigrant cells as determined by RQ-PCR analyses of TRECs were undetectable in patients 1, 2, 3, and 4 and normal in patients 5 and 6 (Table 1). Examination of T-cell receptor V beta region (TCR-V*β*) using FACS (Figures 2(a)–2(g)) revealed a clonal pattern in patients with autoreactive cells (patients 3 and 4, Figures 2(c) and 2(d), resp.). These patients had a clonal pattern with one dominant population (V*β*20) and markedly reduced 20 CD3^+^ V*β*s (patient 3) or two dominant populations (V*β*17 and V*β*7.2), and markedly reduced 17 CD3^+^ V*β*s (patient 4), indicating T-cell clonality. In contrast, both patients with transplacentally acquired maternal lymphocytes displayed skewed oligoclonal patterns in their TCRs (patients 1 and 2, Figures 2(a) and 2(b), resp.). These patients had a restricted pattern with one dominant population (V*β*17) and markedly reduced 8 CD3^+^ V*β*s (patient 1) or two dominant populations (V*β*3 and V*β*12), and markedly reduced 9 CD3^+^ V*β*s (patient 2), indicating T-cell restriction. Since in patient 1 cells were of maternal origin we also examined the V*β* repertoire of this patient's mother and found normal peripheral blood repertoire (Figure 2(g)). TCR-V*β* of both patients with MHC-II deficiency who displayed residual T cells (patients 5 and 6) showed normal polyclonal patterns (Figures 2(e) and 2(f), resp.).

### 3.4. Regulatory T Cells Enumeration and Function

**Figure 3:**
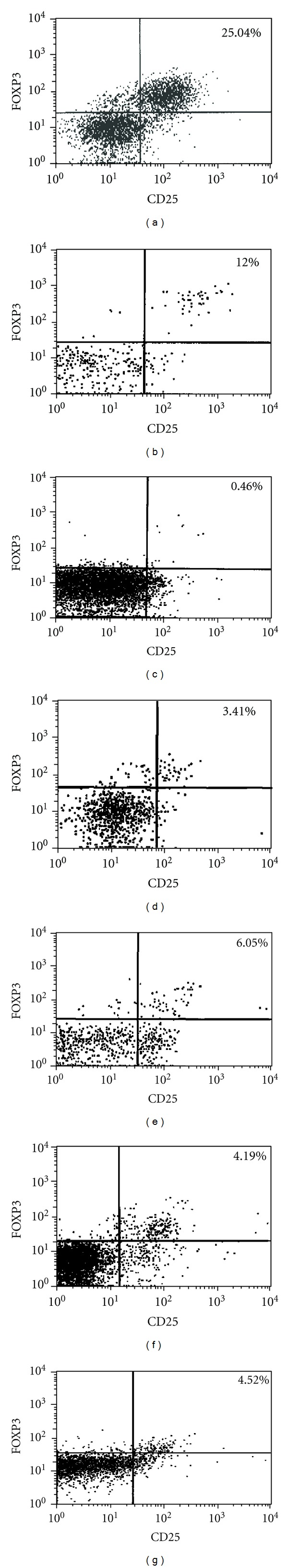
FOXP3 Treg cells in SCID patients. CD25 and FOXP3 expression levels in CD4^+^ T cells of pt1 (a), pt2 (b), pt3 (c), pt4 (d), pt5 (e), age-matched healthy control (f), and the mother of patient 1 (g) were detected using FACS analyses. Quadrants were set up based on staining with isotype control. Boxed numbers indicate the percentage of Treg cells within the CD4^+^ population.

**Figure 4:**
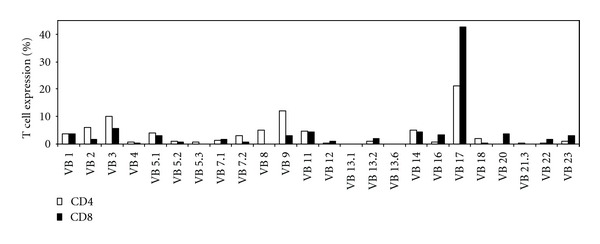
T-cell receptor (TCR) V*β* repertoire. FACS analysis of the relative expression levels of 24 different TCR V*β* families in patient 1 CD3^+^CD4^+^ cells (white bars) and CD3^+^CD8^+^ cells (black bars).

**Figure 5:**
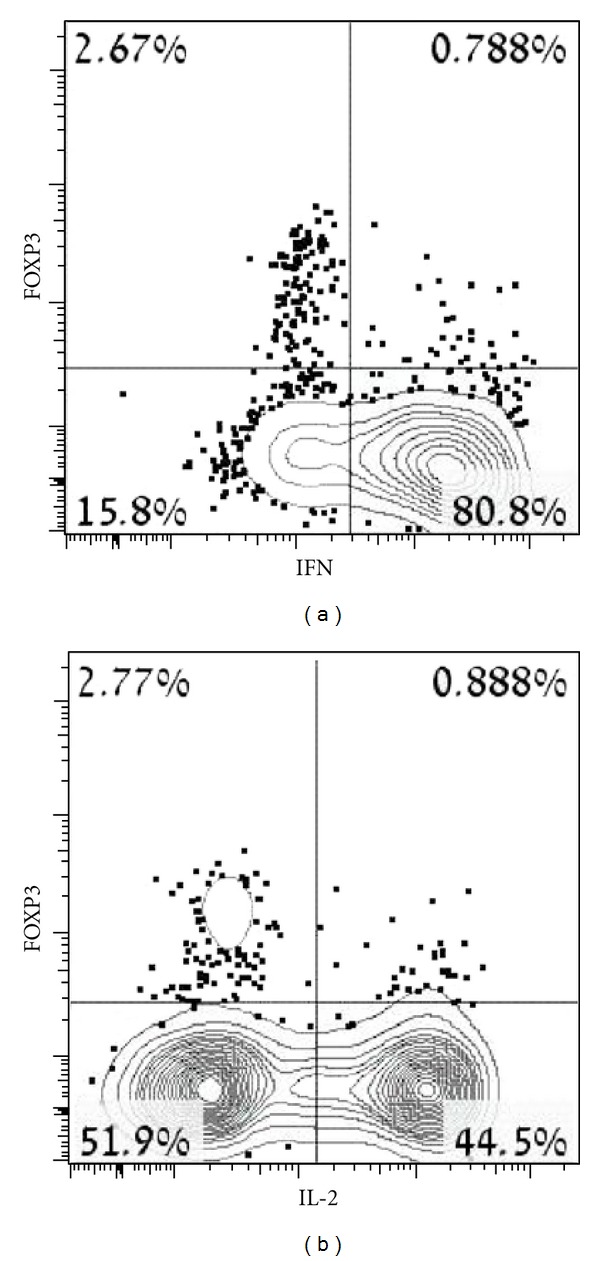
IFN*γ* and IL-2 cytokines secretion following T-cell stimulation. PBMCs obtained from patient 2 were stimulated with PMA and ionomycin, than stained with CD4, FOXP3, and IFN*γ* or IL-2 for the identification of functional Tregs. Detection was performed using FACS analyses. Quadrants were set up based on staining with isotype control. Boxed numbers indicate the percentage of cells within the CD4^+^ population that secrete IFN*γ* or IL-2.

In order to quantify Tregs, unstimulated freshly isolated patients' peripheral blood mononuclear cells (PBMCs) were stained with CD25 and FOXP3 antibodies on live CD4^+^ T cells. Patients 1 and 2 with the alloreactive cells displayed significantly high levels of circulating Tregs (25.4% and 12%, Figures 3(a) and 3(b), resp.). In contrast, low or near normal levels of circulating Tregs were found in patients 3 and 4, containing autoreactive cells (0.46% and 3.41%, Figures 3(c) and 3(d), resp.). Normal levels of Tregs were found in patient 5 who had nonreactive cells (6.05% of total gated cells, Figure 3(e)), compared to age-matched healthy control (4.19%, Figure 3(f)) and to the mother of patient 1 (4.52% of total gated cells, Figure 3(g)). In order to exclude the possibility that the high amount of circulating Tregs in patient 1 overlaps with the cell population showing expended clonality of V*β*17 receptor in this patient (Figure 2(a)), we examined the patient's CD3^+^ V*β*s for CD4 or CD8 expression. The CD3^+^ V*β*17 receptor was composed mainly of CD8^+^ cells (Figure 4) suggesting that the clonal expansion is not composed of Tregs. To examine if the transplacentally acquired Tregs lymphocytes detected in patient 2 are indeed functional, lack of IFN*γ* and IL-2 secretion from these cells was examined. As can be shown in Figure 5, while most of the FOXP3 negative cells produced IFN*γ* and IL-2 cytokines following T-cell stimulation with PMA and ionomycin (80.8% and 44.5% of total CD4^+^ cells, resp.), FOXP3^+^ cells obtained from patient 2 did not secrete IFN*γ* and IL-2 under the same condition, suggesting them as functional Tregs.

## 4. Discussion

Diagnosis of SCID is usually straightforward when patients present with the typical clinical features and a suggestive family history, supported by the results of general immunological tests. The latter includes reduced numbers of the lymphocyte subsets, depressed response of T cells to mitogen or antigen stimulation, and abnormal thymic activity. Immunodeficiency is the hallmark of SCID even in atypical cases where residual or reactive T cells are present. In some of these patients autoimmunity is present as a result of different tolerance mechanisms breakdown. Here we showed that severe immune-deficient patients with circulating T cells display different T-cell functions and regulatory patterns which are in correlation with their T-cell reactivity and the severity of their immunodeficiency. We speculate that some of these immunological parameters can be used to distinct immune deficient patients presenting with residual T lymphocytes of different origins (Table 2). It has been shown that patients with autoreactive cells have profound abnormalities of thymic epithelial cell differentiation and severe reduction of thymic dendritic cells and virtual absence of thymic FOXP3^+^ Tregs [15]. In addition, low thymic and peripheral expression of AIRE and dysfunctional regulatory T cells was demonstrated [13, 16]. Even in cases where individual variability in the fraction of these circulating cells was observed, reduced thymic and lymph node expression of FOXP3 was found. Furthermore, in cases where peripheral FOXP3 expression was demonstrated, it did not identify a *bona fide* natural Treg cell and rather was consistent with an *in vivo* T-cell activation process [17]. Moreover, the expression of FOXP3 does not entirely characterize Tregs in humans and it has also been reported in non-Treg cells. Clinically, these patients will present with autoimmune-like features (e.g., Omenn phenotype). Omenn phenotype was reported in some but not all genetic SCID defects. For example, the defect of MHC class II that leads to combined immunodeficiency with defective CD4^+^ T-cell development and a lack of T-helper-cell-dependent antibody production by B cells, was not reported to cause Omenn phenotype so far [18]. This is probably due to the specific late partial arrest in T-cell maturation that is not necessarily affecting any of the tolerance-regulating mechanisms. In addition, MHC-II-deficient patients are known to have residual T cells with some degree of selected immunity as can be seen in our patients. Interestingly, we found that MHC-II deficient patients have near-normal lymphocyte function and detectable TREC levels. A possible explanation for this finding is the partial T-cell development arrest, and the ability of some residual cells to fully mature in such a deficiency. In addition, no peripheral expansions of T cells are known to occur in these patients that can dilute TREC levels and produce autoimmune features. Partial T-cell development is found also in other SCID variants, such as the common *γ*_c_-R222C hypomorphic mutation, enabling thymic epithelial cell maturation, thymic AIRE expression, and development of FOXP3^+^ T cells [15]. In contrast, as we showed here, patients presenting with reactive T cells (auto- or allo) were found to have severely depressed lymphocyte function and undetectable levels of TRECS. The latter is explained by either because of inability to reach the final stage of T cell maturation or because of a peripheral dilution, secondary to the expansion of T cells that bear no episomal TRECs. Patients with reactive cells, as we showed here, were already been shown to have a restricted TCR repertoire with clonal expansion and autoimmunity [4, 13]. Interestingly, patients with transplacentally acquired maternal T lymphocytes who displayed alloreactive cells present less severe clonal expansion and cell restriction in their circulating CD3^+^ cells compared to the “true Omenn” patients. In addition, they had a high fraction of functional circulating Tregs. Moreover, these cells did not secrete either IFN*γ* or IL-2 cytokines following T cell stimulation, suggesting their ability to suppress autoimmunity. Yet, other assays of Treg function should be used to clarify if indeed these cells are active. The “true” Omenn patients with the autoreactive cells, had low or normal levels of circulating Tregs, as already been shown [17], therefore these patients were suggested to display severe clinical autoimmune phenotype. We speculate that these immunological parameters are able to distinguish between SCID patients presenting with reactive T cells of different origins. While the breakdown of tolerance mechanisms in Omenn may occur simultaneously with the development of autoimmune manifestations, the high tolerance inducing function in some maternal cells allows some, but not all, cells to cross the placenta, survive in the recipient's circulation, and cause mild autoimmunity.

It is well accepted that maternal regulatory T cells mediate maternal tolerance to the fetus in addition to localized mechanisms. Expansion of maternal CD25^+^ T cells with dominant regulatory T-cell activity during pregnancy was observed [19]. We show that these cells continue to express high fractions of the Treg phenotype that might enable better selection and survival. Since these cells are considered to be anergic, secondary expansion of alloreactive clones in patient 1 is unlikely. Moreover, careful analysis of the predominant TCR in our patient (V*β*17, Figure 1) revealed that this clone was composed mainly of CD8-positive cells, and therefore not responsible for the high fraction of the detected Tregs. The maternal cells detected in patients 1 and 2 caused only mild GVHD symptoms, although were HLA mismatched and likely to react with the recipient's organs. Since Tregs are thought to protect against GVHD by inducing and maintaining allogeneic tolerance [20], we then can speculate that the high fraction of circulating Tregs served to balance the immune reaction mediated by the maternal-host dissimilarities, thus protecting against severe GVHD. Indeed, transplacentally acquired maternal T-lymphocytes cells are known to cause only few clinical manifestations, with most cases being entirely asymptomatic, possibly because of the oligoclonal repertoire of the maternal T cells with lack of alloreactivity toward the child's antigens [21]. Yet, the fact that these cells were completely dysfunctional, as evidenced by a lack of response to mitogenic stimulation and the absence of TREC copies, is an indication of poor T-cell differentiation in the thymus that resulted in a severe immunodeficient state. Our study attempts to explain why some patients with SCID and residual T cells present with autoimmunity, and others do not. We provide our data as a speculation since only two patients in each group were studied. Yet, only a small number of patients is expected because of the rarity of these conditions. In summary, our data show that SCID phenotypes with circulating T cells have distinct T cell function, thymic capacity and Treg enumerations which determine their T-cell reactivity and TCR repertoire patterns. Interestingly, transplacentally acquired maternal T lymphocytes in SCID patients have high fraction of functional circulating Tregs but poor T cell differentiation. We speculate that this represents a possible advantage mechanism for their selection over other maternal cells and allows their tolerance by the patient's immune system while still causing a severe immunodeficient state.

## Figures and Tables

**Table 1:** Clinical and immunological findings in 6 patients diagnosed with SCID.

	Pt-1	Pt-2	Pt-3	Pt-4	Pt-5	Pt-6
Diagnosis, genetic defect	SCID-RAG2	SCID-*γ*_*c*_	SCID-RAG2	SCID-RAG2	CID MHC-II	CID MHC-II
Age at diagnosis (months)	5/12	7/12	4/12	3/12	6/12	6/12
Maternal cells	100%	100%	3.5%	2%	0%	0%
Autoimmune features	Mild	Mild	Severe	Severe	No	No
Infections	+	+	+	+	+	+
Lymphocyte count/mm^3^	5600	4500	1320	10686	4900	3416
Eosinophil/mm^3^	1700	600	2960	4030	500	70
CD3/mm^3^	4612	1500	488	2871	3552	1162
CD3^+^CD4^+^/mm^3^	2855	360	244	2351	543	137
CD3^+^CD8^+^/mm^3^	1757	1080	224	855	3305	1009
CD19^+^/mm^3^	1	3015	0	0	543	2186
CD3^−^CD56^+^/mm^3^	504	0	500	3800	490	0
HLADR^+^ (in total lymph)	47%	87%	30%	53%	0%	0%
IGM (IU/mL)	UD	UD	UD	110	UD	UD
IGG (IU/mL)	433	UD	UD	UD	253	UD
IGA (IU/mL)	79	UD	UD	UD	UD	UD
IGE (IU/mL)	UD	UD	UD	UD	UD	UD
PHA mitogenic response*	3.8%	2.4%	6%	6.5%	46.9%	94.8
aCD3 mitogenic response*	1.9%	ND	6.7%	31.5%	41.3%	32.8%
TRECs/0.5 mcg DNA	UD	UD	UD	UD	2769	4384

UD: undetectable, *percentage, CPM patient/CPM control, *γ*_*c*_: common gamma chain, RAG: recombination activating gene.

**Table 2:** Suggested immunological distinctions between SCID patients presenting with residual T lymphocytes of different origins.

Origin of patient's cells	Allo-reactive T cells	Auto-reactive T cells	Residual T cells
SCID phenotype	Transplacentally acquired maternal lymphocytes	Omenn	MHC-II deficiency
Autoimmunity	+	+++	−
Eosinophilia	±	+	−
Lymphocyte count	Normal	Normal	Normal
Lymphocyte subset	Inconsistent, based on the TCR clonality	Inconsistent, based on the TCR clonality	Usually CD4/CD8 reverse ratio
Immunoglobulin levels	Low	Low	Low
Lymphocytes response to mitogens	Low	Low	Normal
TREC	UD	UD	Normal
TCR-V*β* repertoire	Skewed, restricted	Monoclonal	Polyclonal
Treg cells	High	Inconsistent	Normal

UD: undetectable, TCR: T cell receptor, TREC: TCR excision circles, Treg: regulatory T cells.

## Abbreviations

FISH: Fluorescence *in situ* hybridization
FOXP3: Forkhead box P3
GVHD: Graft-versus-host disease
PBMCs: Peripheral blood mononuclear cells
SCID: Severe combined immunodeficiency
TCR V*β*: T-cell receptor variable *β*
TREC: T-cell receptor excision circle
Tregs: Regulatory T cells.
